# Quality of life among men who have sex with men in China measured using the 36-item Short-Form Health Survey: A cross-sectional study

**DOI:** 10.1097/MD.0000000000011310

**Published:** 2018-07-06

**Authors:** Wencheng Zhang, Ping Wang

**Affiliations:** China Medical University, Shenyang, Liaoning Province, China.

**Keywords:** associated factor, men who have sex with men, quality of life, Short-Form Health Survey

## Abstract

The present study was conducted to assess the quality of life (QOL) of Chinese men who have sex with men (MSM) and to explore possible factors associated with QOL among them.

A cross-sectional study of 370 MSM in Dalian and Huludao city was conducted to evaluate QOL in MSM using the Chinese version of the 36-item Short-Form Health Survey (SF-36). A *t* test was used to compare the QOL score of our sample with the Chinese norm. ANOVA, *t* test, and multivariate linear regression analysis were used to assess the association of QOL with basic characteristics.

The QOL score of MSM was lower than the Chinese norm (*P* < .05). Multivariate analysis showed that marital status and monthly income were factors associated with physical component summary (PCS) and mental component summary (MCS) among MSM population (*P* < .05). Married MSM had poorer QOL, and respondents with higher income levels had better QOL.

The government may need to develop more effective prevention strategies to improve the mental component of QOL in the Chinese MSM population, especially the married MSM population.

## 1 Introduction

Sexual transmission, especially male-to-male homosexual transmission, has become one of the main modes of HIV transmission since 2007 in China.^[1]^ Moreover, men who have sex with men (MSM) may also be responsible for spreading HIV from high-risk male sexual partners to lower-risk female partners.^[2]^ The HIV infection rate for MSM has increased; MSM accounted for 0.2% of new infections in China in 2001, 12.2% in 2007, and 32.5% in 2009.^[3]^ The HIV prevalence among Chinese MSM in 2011 was 6.3%, which was about 5 times that in 2001.^[4]^ MSM have become a population of concern in terms of preventing HIV transmission.^[5]^

As a hidden population in China, it is a significant challenge to reach the MSM population, in which prevention strategies may not fulfill the goal of reducing new infections.^[6]^ Some studies showed that life stress associated with poor physical and mental status could make MSM avoid seeking HIV tests, which could make them vulnerable to HIV infection.^[7–9]^ Interventions focused on improving mental health and structural interventions targeting physiological and safety concerns could make MSM more willing to accept public health services.^[9]^ Eriksson et al^[10]^ studied the quality of life (QOL) among the MSM and provided additional information about the influencing factors that made them interested in availing counseling and care.

Through analyzing QOL among Chinese MSM, we could understand the specific defects of their mental and physical health, and determine the factors related to QOL. These will potentially provide information about targeted interventions on improving the QOL among MSM, which could increase survival and decrease health care costs due to HIV disease progression. The 36-item Short-Form Health Survey (SF-36) is a concise tool that can be used to assess QOL, and has wide cross-cultural compatibility. Globally, it has become a widely used tool for QOL evaluation.^[11]^ SF-36 has proven to be useful in monitoring population health, assessing the burdens of different diseases, and evaluating the effects of medical treatment.^[11]^ It also has been used successfully for assessing the QOL of HIV-infected MSM.^[12]^ The SF-36 not only provides a direct quantitative indication of the condition of physical health, but also provides an indication of the state of mental health.^[12–14]^

In this study, our aim was to critically assess the QOL, and to explore the factors associated with QOL among Chinese MSM, in order to provide information for prevention strategies.

## 2 Methods

### 2.1 Study samples and procedures

From April 2016 to September 2016, a cross-sectional survey was conducted in Dalian and Huludao City, China. The study sample was from venues such as gay saunas, gay bars, and parks where MSM meet one another. The enrollment criteria were as follows: male, 18 years of age or older, reported having sex with male in the past year, and willing to finish the study.

All eligible participants completed the self-administered questionnaire in a private room in the local Center for Disease Control and Prevention (CDC). Participation in the study was completely voluntary, and respondents could decline to answer any questions if they did not wish to participate. In total, 377 eligible subjects were included in this survey, and 370 completed questionnaires were returned (response rate 98.1%).

### 2.2 Measures

The questionnaire consisted of 2 parts: the basic information and SF-36. The basic information form included socio-demographic information (age, marital status, monthly income, education, and vocation) and sleep quality. The basic information data were collected using a self-designed questionnaire consisting of items assessing the demographics of interest. The item “is your sleep quality good” in a “yes or no” format was used to evaluate the sleep quality.

The SF-36 includes 36 items covering 8 domains: physical function (PF, 10 items), role-physical (RP, 4 items), bodily pain (BP, 2 items), general health (GH, 5 items), vitality (VT, 4 items), social function (SF, 2 items), role-emotional (RE, 3 items), and mental health (MH, 5 items). The Physical Component Summary (PCS) is assessed by grouping all physical components (PF, RP, BP, and GH) together; similarly, the Mental Component Summary (MCS) encompasses mental components (VT, SF, RE, and MH). Each domain and component summary is scored from 0 to 100, with higher scores indicating better QOL. The reliability of SF-36 in this study was satisfactory, with the Cronbach alpha equal to 0.96.

### 2.3 Statistical analysis

The comparison of QOL score between our sample and the Chinese norm^[15]^ was analyzed by the *t* test. ANOVA, *t* test, and multivariate linear regression analysis were performed to assess the association between QOL and related factors. All variables found to be significant by *t* test and ANOVA analysis were included in a multivariate linear regression model with stepwise procedure. The data were analyzed using SPSS version 17.0 (SPSS Inc., Chicago, IL) for Windows. A *P*-value of < .05 was considered statistically significant.

### 2.4 Ethical review

The study was approved by the bioethics advisory commission of China Medical University. Before participating in the study, participants were informed about the purpose of the study and were assured that their privacy would be protected. Verbal informed consent was obtained from each participant before study enrollment.

## 3 Results

### 3.1 Basic characteristics of the participants

A total of 370 men completed the survey. The average age of respondents was 27.67 ± 8.62 years (ranged from 18 to 69 years). Of all participants, 10.8% were below 20 years of age, 73.0% were single, and 80.3% respondents graduated from senior high school education or more. Thirty-one percent (117) of MSM reported that their sleep quality was not good. The basic characteristics are shown in Table 1.

**Table 1:**
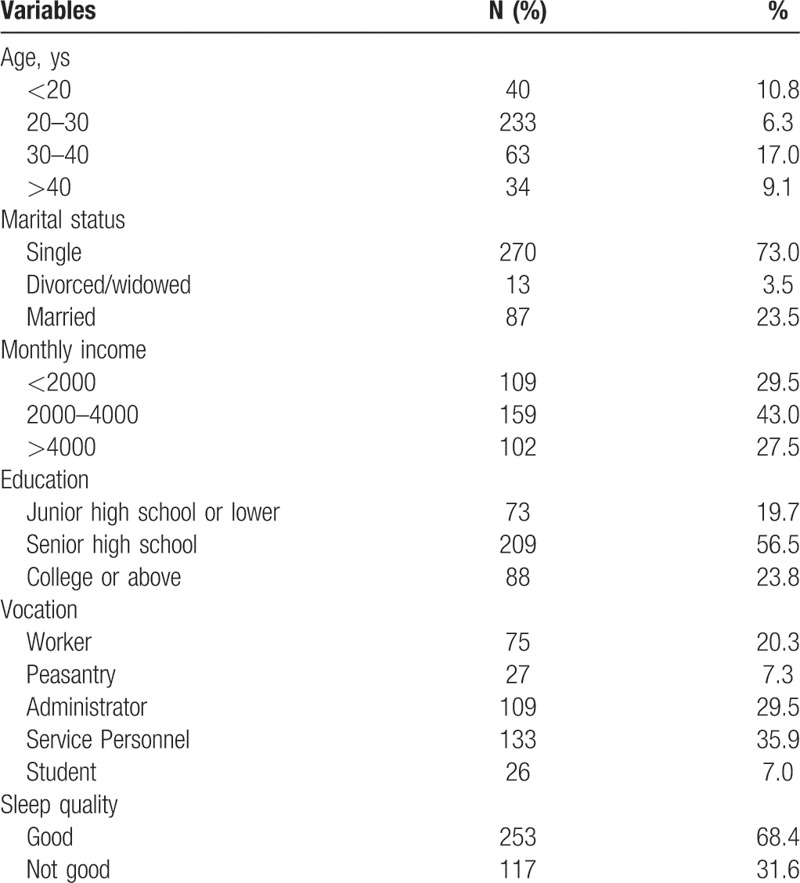
Basic characteristics of study sample (N = 370).

### 3.2 QOL of the participants

The mean score for PCS (73.09 ± 11.07) was higher than for MCS (63.86 ± 17.52). Participants scored highest for BP (84.15 ± 16.09) and lowest for MH score (56.40 ± 13.73). When comparing results between scores among MSM and the Chinese norm, scores on all 7 domains (with the exception of the BP domain)^[15]^ were significantly lower in MSM than the Chinese norm (*P* < .05) (Table 2).

**Table 2:**
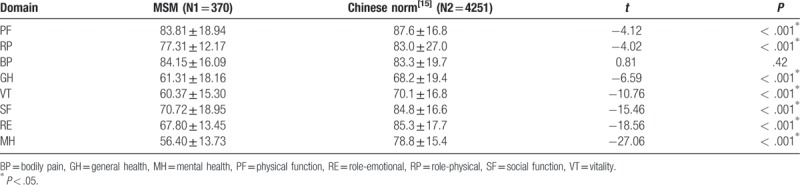
Comparison of quality of life among men who have sex with men with Chinese norm.

### 3.3 Factors associated with physical components of QOL among MSM

Univariate analysis showed that there were significant differences in PCS score between different age groups, marital status, monthly income, and sleep quality (*P* < .05). All of the 4 domain scores (PF, RP, BP, and GH) were significantly different for the group of different marital status. The married MSM got lower scores. The results are shown in Table 3.

**Table 3:**
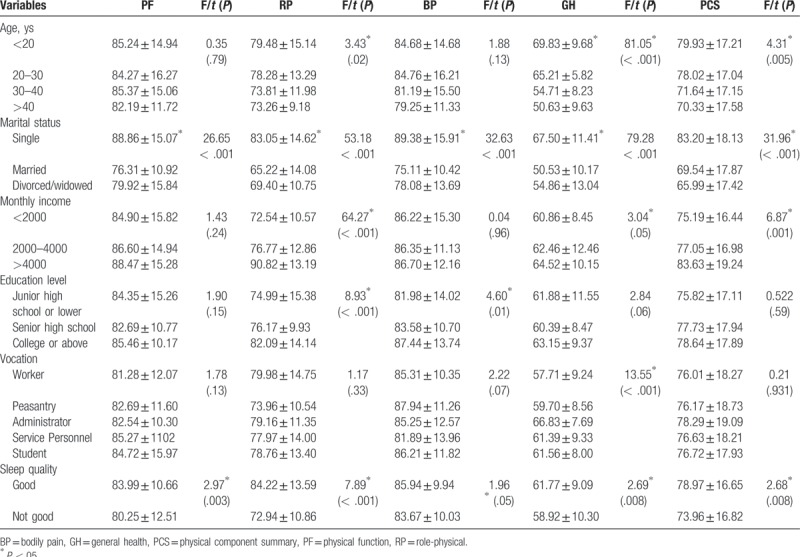
Factors associated with physical component of quality of life among men who have sex with men (N = 370).

### 3.4 Factors associated with mental components of QOL among MSM

Univariate analysis (Table 4) revealed that there were significant differences in MCS scores between those with a different marital status, monthly income, education, vocation, and sleep quality (*P* < .05). All the 4 domain scores (VT, SF, RE, and MH) showed significant differences based on marital status, vocation, monthly income, and sleep quality. The married MSM had lower scores, and participants who reported good sleep quality had better scores. Compared with other types of vocation, those involved in peasantry had the lowest scores. The QOL scores were also positively associated with income (*P* < .05).

**Table 4:**
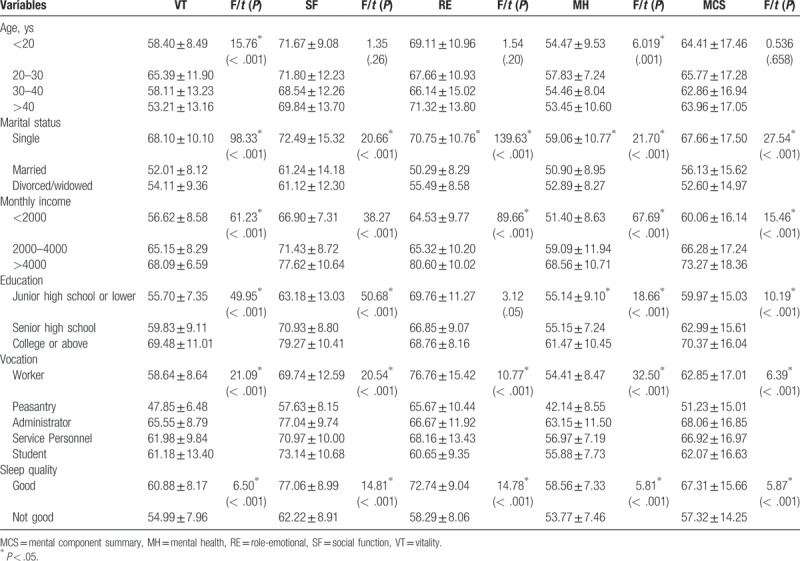
Factors associated with mental component of quality of life among men who have sex with men (N = 370).

### 3.5 Factors associated with QOL among MSM in the multivariate analysis

Multivariate linear regression analysis was used to identify factors related to QOL of the Chinese MSM population. Significant associated factors of PCS included in the model were marital status and monthly income (*P* < .05). The married MSM had lower score of PCS. Monthly income was positively associated with PCS score (*P* < .05), with a coefficient of 0.13. For MCS, sleep quality, marital status, and monthly income were significant associated factors (*P* < .05). The married MSM and respondents with bad sleep quality had lower scores of MCS (*P* < .05). Scores increased with income levels, and the coefficient was 0.14 (*P* < .05) (Table 5).

**Table 5:**
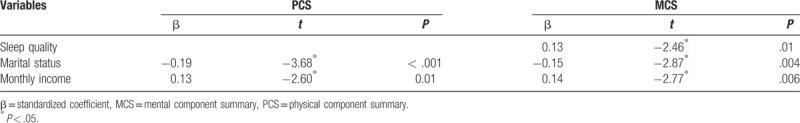
The multivariate linear analysis for identifying the associated factors of quality of life among men who have sex with men (N = 370).

## 4 Discussion

The QOL scores of MSM in this study were lower than the Chinese norm (*P* < .05), and the mental components of QOL was poorer than the physical aspect. Accumulating data indicate that due to discrimination, mental health problems are more prevalent among sexual minorities than among heterosexuals.^[16,17]^ In one population-based survey of the US adults, 21.4% of lesbian, gay, or bisexual participants reported experiencing discrimination in the past year.^[18]^ In China, compared with the general population, MSM had higher levels of depression and anxiety symptoms.^[19]^ Homosexuality remains subject to discrimination because of society's emphasis on procreation and social order.^[20,21]^

Both the results of univariate and multivariate analyses suggested that marital status was an important associated factor of QOL in the MSM population. Our study found that married MSM had the lowest scores in all eight domains and both component summaries. Charles et al^[22]^ suggested that married people living with HIV in India may be afraid of disclosing their status to their family owing to the fear of losing social and economic support, which in turn caused them to experience serious depression, anxiety, and social isolation. Marital decisions among MSM are also affected by Chinese cultural norms that place pressures on sons to marry. Marital pressures make MSM have to reconcile same-sex attractions with filial expectations.^[21]^ In order to avoid social pressure, most Chinese MSM marry heterosexual women, which may cause married MSM to not only experience pressure from social discrimination associated with homosexuality but also experience pressure from their spouse and family.^[23]^ It was suggested that interventions should find ways to help MSM navigate a balance between their own needs and the responsibilities they feel toward their family.^[21]^ Therefore, more attention needs to be paid to the QOL of married MSM.

For the respondents in our study, a lower monthly income was related with worsened QOL, which was consistent with other studies on people living with HIV.^[24,25]^ Ling Huang et al^[26]^ found that lack of money may lead to poor nutrition and a lack of medical care, which could account for poor physical conditions. Previous research suggested that lower income was associated with poorer mental health among HIV-positive gay men.^[27]^ According to the results of univariate analysis, we found that the scores of the physical component of QOL (GH, VT, and PCS) showed a decline with increasing age. With the increase in age, people's physical function and the ability to participate in social activities declined.^[28,29]^

Our study showed that respondents with a junior high school or lower education level had the lowest scores in the mental component of QOL (VT, MH, SF, and MCS). As reported in Brazil, lower education levels were associated with deteriorating QOL in people living with HIV.^[30]^ Results from Spain and China showed that people with lower education levels had more stress and a worse score on the mental component of QOL than did respondents with higher education levels.^[29,31]^ Another study concluded that in China, higher education levels may enable people to have more knowledge on how to deal with pressure.^[32]^

There were significant differences in several domains between the different vocational subgroups. Comparing to other vocations, men who had peasantry as a vocation had the lowest scores in QOL, especially in the mental component of QOL (MH, VT, SF, and MCS). In our country, the persons who are involved in peasantry live in very traditional rural communities where homosexuality may be less understood than in urban areas. In addition they have very a few sources of entertainment and enjoyment.^[33]^ Difficult living conditions and social ignorance could contribute to the bad mental status of the peasantry among the MSM population of China. In comparison, MSM in rural communities in South Africa do not yet receive enough attention from the public health sector and social services.^[34]^

There are some limitations in this study. First, the study was conducted in only 2 Chinese cities, which will limit the representation of Chinese MSM. Thus, it may have been more ideal to survey a larger, more representative sample in order to better generalize results for all Chinese MSM. Second, the unique use of self-report response may cause some bias, such as consistency motifs, acquiescence bias, and social desirability. Lastly, this study was a pilot observational investigation employing a cross-sectional design to explore the factors associated with QOL among MSM. In further studies, focusing on the association of behavioral factors with QOL and applying a more effective study design should be considered to investigate the factors associated with QOL among Chinese MSM.

## 5 Conclusion

The QOL among the Chinese MSM population was poorer than that among the general population, especially when the mental component of QOL was considered. Marital status was an important factor associated with QOL, as married MSM had poorer QOL. The poor mental component of QOL among MSM in China must be improved, and action from the public health community is required. Government prevention strategies should pay special attention to the married MSM population.

## Acknowledgments

The authors wish to thank all the MSM participants in this study.

## Author contributions

**Conceptualization:** Wencheng Zhang.

**Data curation:** Wencheng Zhang.

**Formal analysis:** Wencheng Zhang.

**Funding acquisition:** Wencheng Zhang.

**Investigation:** Wencheng Zhang.

**Methodology:** Wencheng Zhang.

**Project administration:** Wencheng Zhang.

**Resources:** Wencheng Zhang.

**Software:** Wencheng Zhang.

**Supervision:** Wencheng Zhang, Ping Wang.

**Validation:** Wencheng Zhang.

**Visualization:** Wencheng Zhang.

**Writing – original draft:** Wencheng Zhang.

**Writing – review & editing:** Wencheng Zhang.
